# Determinants of out-of-pocket expenditure on medicines among adults in Saudi Arabia: a cross-sectional study

**DOI:** 10.3389/fmed.2024.1478412

**Published:** 2024-11-08

**Authors:** Mohammed Khaled Al-Hanawi, Mpho Keetile

**Affiliations:** ^1^Department of Health Services and Hospitals Administration, Faculty of Economics and Administration, King Abdulaziz University, Jeddah, Saudi Arabia; ^2^Health Economics Research Group, King Abdulaziz University, Jeddah, Saudi Arabia; ^3^Department of Population Studies, University of Botswana, Gaborone, Botswana

**Keywords:** determinants, expenditure, out-of-pocket, medicines, Saudi Arabia

## Abstract

**Introduction:**

To achieve universal health coverage consistent with World Health Organization recommendations, monitoring financial protection is vital, even in the context of free medical care. Toward this end, this study investigated out-of-pocket (OOP) expenditure on medicines and their determinants among adults in Saudi Arabia.

**Methods:**

This analysis was based on cross-sectional data derived from the Family Health Survey conducted by the General Authority for Statistics in 2018. Data analyses for this study were based on the total sample of 10,785 respondents. Descriptive statistics were used to identify the sample distribution for all variables included in the study. Tobit regression analysis was used to examine the determinants of OOP expenditure on medicines.

**Results:**

The average OOP expenditure on medicines was estimated to be 279.69 Saudi Riyal in the sampled population. Tobit regression analysis showed that age, average household monthly income, education level, and suffering a chronic condition were the main determinants of OOP expenditure on medicines. Conversely, being married and employed were associated with a lower probability of OOP expenditure on medicines.

**Conclusion:**

This study could assist policy makers to provide additional insurance funding and benefits to reduce the possibility of catastrophic OOP expenditure on medicines, especially for the most vulnerable demographic.

## Introduction

There has generally been a global improvement in average household income and life expectancy in recent decades related to improvements in healthcare outcomes. Despite general improvements in quality of life in the general population, the opposite trend is evident for the older adult population (1). Most developed countries are experiencing an increase in the proportion of the older adult population, which is often associated with an increased prevalence of chronic non-communicable diseases and complex medical conditions, resulting in an increased demand for healthcare services, including access to medicines (2). Inequalities in medical supplies and resource distribution are likely to further affect the most socioeconomically disadvantaged populations (3, 4). The Organization for Economic Co-operation and Development (OECD) projected that adult populations will significantly influence public expenditures, indicating that long-term care and health will comprise over half the rise in age-related societal costs by 2050. The increasing costs of medicines and general care may result in catastrophic health expenditure (CHE) and poverty in the near future (5–7). CHE occurs when a family’s health expenditure is equal to or more than 40% of the total family expenditure after subtracting subsistence costs (8, 9).

Out-of-pocket (OOP) expenditures for healthcare utilization, medicines, and medical services of older adults are all higher than those of the general population (10–12). However, these data are based on studies from high-income countries (HICs), with less focus on the situation in low-and middle-income countries (LMICs) (13, 14). Moreover, relative OOP expenditure as a share of total spending on medicines among OECD member states (41% in 2011) was more than double that spent on healthcare services (18% in 2011) (15). The limited available evidence from LMICs corroborates these findings (12–14, 16).

It is estimated that over 150 million people are impacted by the economic burden of OOP health expenditure and that approximately 100 million people reach a state of poverty due to OOP medical and healthcare expenditures worldwide (17, 18), with the greatest experience by low-income families (19, 20). To provide protection against the adverse effects of OOP health expenditure, the World Health Assembly resolution recommended that member states should target the achievement of universal health coverage (UHC) (21) to ensure equal access to healthcare services utilization, especially when required, with no financial barriers. Achieving this goal involves multi-faceted strategies, including targeting the proportion of medical costs, variety of services, and number of people covered (22).

Given that the pharmaceutical market in the Kingdom of Saudi Arabia (KSA) is the largest, most volatile, and most sophisticated in the Middle East and North Africa region, understanding the determinants of OOP expenditure on medicines in the KSA is vitally important. The majority of medicines provided to Saudi citizens are paid for by the government, while non-Saudi nationals are covered by mandatory private health insurance provided by employers (22). Individuals who use private health insurance are generally treated in the private sector. Since the KSA offers universal primary healthcare to the population, it is generally assumed that OOP expenditure on medicines and healthcare should be nominal. However, the most recent data suggest that retail sales account for 44% of medicine in terms of value (23).

Although the KSA is considered to be an HIC, it also has a notable low-income population (24–26). The provision of free healthcare services to its citizens imposes pressure on public health facilities that ultimately affects public healthcare provision. Some studies have demonstrated notable variation in OOP expenditure on medicines and health in general, even in the context of UHC for primary healthcare (19, 27, 28). The pharmaceutical industry and health financing system are evolving rapidly in the KSA (22, 23). Therefore, understanding the determinants of OOP expenditure on medicines is important for policy interventions.

The few studies on OOP health expenditure in the KSA focused on OOP expenditure for healthcare, health insurance, and chronic conditions to provide insight into the total or public resources devoted to health (10, 22, 29, 30); however, the determinants of OOP expenditure on prescribed medicines remain unclear. This may be due to the lack of adequate routine data on pharmaceutical expenditures collected at the individual and household levels.

To address this gap, this study investigated inequalities in OOP expenditure on medicines and their determinants among adults in the KSA based on data from a large national survey. Identifying the population with the highest risk of facing CHE and the consequent economic behaviors can guide policymakers to better target the distribution of public health insurance.

## Methodology

### Study design

This was a cross-sectional analysis of self-weighted data obtained from the 2018 Family Health Survey (FHS) (31), conducted by the General Authority for Statistics (GaStat) in Saudi Arabia in collaboration with various entities in the health sector of Saudi Arabia, including the Ministry of Health, Saudi Health Council, private organizations, and academic institutions.

### Population and setting

The FHS is carried out every 3 years and falls under the classification of education and health statistics. The FHS collects information by visiting a representative sample of the population across all 13 administrative regions of Saudi Arabia.

Given its extensive coverage of health-related information and its representative nature, the FHS dataset was well-suited for examining the heterogeneous relationship between OOP expenditure on medicines and various explanatory variables.

### Sample

The total sample size for the FHS was 15,265 responses, randomly selected from the 13 administrative regions of Saudi Arabia. Our analysis was limited to respondents who were aged 18 years or older and had completed information on all the variables of interest. After excluding those with missing responses to healthcare-related questions and covariates, the final sample consisted of 10,785 respondents.

### Outcome variable

To examine the determinants of OOP expenditure on medicines in the KSA, the outcome variable was taken from a section of the FHS on health status where respondents were asked to report their average household monthly OOP expenditure on medicines prescribed by a healthcare professional and their average household monthly income (in Saudi Riyal [SR]). This is a continuous variable reported in SR (1 US$ = 3.75 SR). As a result, analysis of expenditures on non-prescribed medications is beyond the scope of this study.

### Explanatory variables

The survey also contains rich information on demographic and socioeconomic status. Among these, the selection of explanatory variables used in this study was derived from the existing literature on the determinants of OOP expenditure on medicines (32). Table 1 shows the list of the independent variables that could influence OOP expenditure in medicine, their explanation, measurement and categorization, and the expected direction of regression coefficients in relation to OOP expenditure in medicines.

**Table 1:** Independent variable specifications.

Variables	Explanation	Measurement and categorization	Expected direction in relation to OOP expenditure on medicines
Average monthly income	Average household monthly income	Continuous, in SR	(+)Those earning higher incomes are expected to be able to afford to spend more on prescribed medicines
Gender	Whether the respondents are men or women	0 = women1 = men	(+)Men are more likely to incur OOP expenditure on medicines because they are usually the main income earners
Age	Age of respondent	Continuous, in years	(+)Elderly people are expected to incur more OOP expenditure on medicines as they are more likely to suffer from aging-related diseases
Marital status	The marital status of the respondent	0 = unmarried^*^1 = married	(+/−)Not clear
Education level	The highest education level respondent obtained	0 = below primary school1 = primary school2 = intermediate school3 = secondary school4 = pre-university diploma5 = higher education (university degree or/and postgraduate degrees)	(+)People with a higher education level are expected to be more likely to pay for medicines because more education increases the level of understanding and awareness of the importance of the medicines
Employment status	The employment status of the respondents	0 = unemployed^**^1 = employed2 = retired	(+/−)Not clear
Insurance status	Whether the respondent has private health insurance	0 = uninsured1 = insured	(+/−)Not clear
Nationality	The nationality of the respondents	0 = non-Saudi1 = Saudi	(+/−)Not clear
Health status level(self-rated health)	The health status level of respondents	0 = very bad or bad1 = mediocre2 = good or very good	(+)Individuals who rate their health status as ‘bad or very bad’ would have higher OOP expenditure on medicines
Chronic condition	Whether the respondent suffers from a chronic disease	0 = not suffering from a chronic condition1 = suffering from a chronic condition	(+)Those who are suffering from a chronic condition are more likely to incur OOP expenditure on medicines

* Unmarried includes single, divorced, and widowed categories.

** Unemployed includes unemployed, those enrolled in education or training, and those dedicated to the work of the house.

The explanatory variables tested included personal, household, and health-related characteristics. Sum et al. (33) reported that personal characteristics such as gender, age, education, marital status, employment status, and nationality are very important predictors of whether or not individuals will utilize medicines. Income and educational level were found to be among the most important predictors of OOP expenditure on medicines in Eastern Asia (34).

Economic status, as measured by average household income, has been highlighted as a strong determinant of OOP expenditure on medicines (35). The same pattern has been shown in previous studies in the KSA, demonstrating a significant association of household income with OOP health expenditure in general (26). Therefore, we included monthly household income as a proxy for economic status in this study as this variable is reflective of the household’s overall wealth status (36).

We expected that higher monthly income would be positively correlated with the amount of OOP expenditure on medicines. Thus, we anticipated that households with higher monthly incomes would be able to afford to spend more on prescribed medicines compared to households with low monthly incomes. Indeed, households with low monthly incomes are more likely to spend a relatively larger portion of their budget on medicines compared to counterpart households with higher monthly incomes. Moreover, households with higher monthly incomes are characteristically wealthier, often have access to information, and therefore have better knowledge about healthcare (37). For example, they may know where to buy inexpensive medicines and where to obtain good price discounts for medicines. Consequently, household income was hypothesized to influence OOP expenditure on medicines.

Other important characteristics affecting the expenditures and utilization of medicines include health-related variables such as suffering from a chronic disease, health status level, and health insurance coverage. Suffering from a chronic disease was set as a dummy variable, which was hypothesized to be linked to the OOP expenditure on medicines. Having members in the household who suffer from chronic diseases requires more resources to be devoted to their care. Chronic conditions such as hypertension or diabetes require lifelong medication and therefore pose a heavy burden (38). Moreover, we hypothesized that individuals who rate their health status as ‘bad or very bad’ would have higher OOP expenditure on medicines. In other words, households with people who have poor health will need to spend more on the prescribed medications, thereby significantly increasing their vulnerability to poverty (39).

Although health insurance is opined to reduce OOP expenditure on medicines, the evidence on this association is at best mixed. While there is some evidence to suggest that health insurance indeed reduces OOP expenditure (40, 41), other studies indicate that health insurance coverage increases OOP expenditure (42–44). These contradictions invite the question of interrogating whether health insurance coverage reduces or increases OOP expenditure on medicines in the KSA. Although the KSA is categorized as an HIC, it has some attributes of LMICs (24), which make its context comparable to the related literature for both categories. Consequently, exploration of the link between insurance coverage and OOP expenditure on medicines in the KSA could help to explain the heterogeneous relationships reported in the literature to date.

### Statistical analyses

Data analyses for this study were based on the total sample of 10,785 respondents. We used descriptive statistics to identify the sample distribution for all variables included in the study. Percentage, mean, and standard deviation (SD) values for OOP expenditure on medicines (our primary outcome) were calculated to describe the sample.

Tobit regression analysis (45) was used to identify the determinants of OOP expenditure on medicines prescribed by a healthcare professional, although ordinary least squares (OLS) multiple regression is typically used when continuous data are obtained from an open-ended question. However, the large number of survey responses of zero OOP expenditure on medicines raises concern about the continuity of the dependent variable. In addition, the OLS estimation fails to account for the qualitative differences between the limit observations (zero OOP expenditure on medicines) and non-limit observations (positive OOP expenditure on medicines) (46). This can lead to biased and inconsistent estimation of the marginal effects (47). It has been argued that the nature of the OOP expenditure question is continuous with censoring at zero. Therefore, the use of a standard linear regression model (48) with the Tobit model is deemed a more appropriate estimation technique in this context of such a limited dependent variable (46).

Previous studies have also indicated that the Tobit model is suitable for analyzing OOP expenditure when the nature of this variable is continuous with censoring at zero (44, 49–51). Consequently, Tobit regression analysis was used in this study to estimate the ‘beta’ coefficients associated with OOP expenditure on medicines in the KSA and examine how OOP expenditure on medicines varies based on respondents’ socioeconomic characteristics. The marginal effects, denoted as *β*′ and *β*″, were estimated. *β*″ represents the marginal effects for the probability of being uncensored, while *β*″ represents the marginal effects for the expected OOP expenditure on medicines conditional on being uncensored: E (OOP on medicines | OOP on medicines >0) (52, 53). All data analyses were performed using STATA SE 14 (StataCorp LP, TX, USA).

## Results

### Descriptive analysis

Table 2 shows the socioeconomic characteristics of the sample. The sample constituted a high proportion of men (54.3%), those who were married (68.5%), with secondary school education (28.9%), unemployed (47.1%), Saudi nationals (74.3%), those self-reporting a good or very good health status (84.6%), those not covered by health insurance (67.3%), and those who were not suffering from a chronic condition (66.0%).

**Table 2:** Characteristics of the sample.

Variable	Mean or *N*	SD or %
Average household expenditure on medicines (Saudi Riyal [SR]), mean (SD)	279.69	749.97
Average household monthly income (SR), mean (SD)	10,648.19	10,106.58
Age, mean (SD)	42.40	16.96
Gender, *N* (%)		
Women	4,929	45.7
Men	5,856	54.3
Marital status, *N* (%)		
Unmarried	3,394	31.5
Married	7,391	68.5
Education level, *N* (%)		
Below primary school	2,213	20.5
Primary school	1,128	10.5
Intermediate school	1,325	12.3
Secondary school	3,118	28.9
Pre-university diploma	709	6.6
Higher education	2,292	21.3
Employment status, *N* (%)		
Unemployed	5,076	47.1
Employed	4,368	40.5
Retired	1,341	12.4
Insurance status, *N* (%)		
Not insured	7,253	67.3
Insured	3,532	32.7
Nationality, *N* (%)		
Non-Saudi	2,772	25.7
Saudi	8,013	74.3
Self-rated health, *N* (%)		
Very bad or bad	494	4.6
Mediocre	1,167	10.8
Good or very good	9,124	84.6
Chronic condition, *N* (%)		
Not suffering from a chronic condition	7,121	66.0
Suffering from a chronic condition	3,664	34.0

SD, standard deviation.

### Determinants of OOP expenditure on medicines

The results of the Tobit regression analysis are presented in Table 3, and the marginal effects are presented in Table 4. In accordance with *a priori* expectations, average household monthly income was significantly associated with OOP expenditure on medicines, suggesting that individuals earning higher income had a higher probability of spending OOP on medicines (*p* < 0.01). Moreover, the education variable also had a positive coefficient in the Tobit regression. This suggests the probability that an individual with a secondary school level of education and higher education level of education would have, respectively, 0.06 and 0.08 greater OOP expenditure on medicines than individuals with an education level of primary school or below. Moreover, those with secondary school education and those with higher education spent approximately 53 SR and 72 SR more, respectively, OOP on medicines than those with an education level below primary school. All these results were significant at a *p*-value of <0.001.

**Table 3:** Tobit regression analysis on factors influencing OOP expenditure on medicines.

Explanatory variable	*B*	(*B* S.E.)
Average household monthly income	0.034***	(0.006)
Age	1.686*	(0.955)
Gender		
Women	(Ref)	
Men	−26.168	(25.642)
Marital status		
Unmarried	(Ref)	
Married	−41.772**	(19.774)
Education level		
Below primary school	(Ref)	
Primary school	117.982***	(27.947)
Intermediate school	179.555***	(28.500)
Secondary school	137.395***	(29.081)
Pre-university diploma	237.997***	(46.156)
Higher education	182.780***	(35.697)
Employment status		
Unemployed	(Ref)	
Employed	−89.076***	(29.676)
Retired	−62.843	(43.084)
Insurance status		
Not insured	(Ref)	
Insured	−10.049	(19.737)
Nationality		
Non-Saudi	(Ref)	
Saudi	24.046	(27.631)
Self-rated health		
Very bad or bad	(Ref)	
Mediocre	73.932	(42.029)
Good or very good	−15.110	(42.612)
Chronic condition		
Not suffering from a chronic condition	(Ref)	
Suffering from a chronic condition	186.358***	(33.904)

Ref, reference category; *p < 0.10; **p < 0.05; ***p < 0.01.

**Table 4:** Marginal effects of factors influencing OOP expenditure on medicines.

Explanatory variable	*β′*	*β″*
Average household monthly income	0.001***	0.013***
Age	0.001*	0.638*
Gender		
Women	(Ref)	
Men	−0.012	−9.907
Marital status		
Unmarried	(Ref)	
Married	−0.019**	−15.889**
Education level		
Below primary school	(Ref)	
Primary school	0.054***	46.125***
Intermediate school	0.083***	71.255***
Secondary school	0.063***	53.061***
Pre-university diploma	0.109***	96.845***
Higher education	0.084***	71.775***
Employment status		
Unemployed	(Ref)	
Employed	−0.041***	−33.504***
Retired	−0.029	−23.386
Insurance status		
Not insured	(Ref)	
Insured	−0.004	−3.797
Nationality		
Non-Saudi	(Ref)	
Saudi	0.011	9.059
Self-rated health		
Very bad or bad	(Ref)	
Mediocre	0.034	28.545
Good or very good	−0.007	−5.737
Chronic condition		
Not suffering from a chronic condition	(Ref)	
Suffering from a chronic condition	0.086***	72.034***

*β*′ represents the marginal effects for the probability of being uncensored, and *β*″ represents the marginal effects for the expected OOP expenditure on medicines value conditional on being uncensored: *E* (OOP expenditure on medicines | OOP expenditure on medicines > 0). Ref, reference category; *p < 0.10; **p < 0.05; ***p < 0.01.

Moreover, age emerged as a significant factor contributing to OOP expenditures on medicine at a *p*-value of <0.1. This suggests that as age increases, the probability of spending OOP on medicines increases. Suffering from a chronic condition was also found to be significantly associated with OOP expenditure on medicines at a *p*-value of <0.01. Specifically, those suffering from a chronic condition had a 0.08 greater probability of spending OOP on medicines than those not suffering from a chronic condition, spending approximately 72 SR more than their counterparts. Conversely, marital status and employment status had negative coefficients in the Tobit model (Table 4), suggesting that married and employed individuals have an approximately 0.02 lower probability of spending OOP on medicines with significance at the 0.05 and 0.01 level, respectively.

## Discussion

The findings of this study indicate that the sampled respondents paid an average of 279.69 SR OOP for medicines, even though Saudi Arabia provides free healthcare services to the general public (54). However, this finding is not surprising since some previous studies have shown that free public healthcare usually creates pressure on public health facilities and affects public healthcare provision (24, 25). As a result, some people, especially public sector employees, ultimately go to private health facilities and pay for prescribed medicines OOP, while others prefer the option of purchasing private health insurance packages to safeguard their income and wealth against the high prices of some medicines.

Age was found to be positively associated with OOP expenditure in medicines, suggesting that as age increases, the probability of spending OOP on medicines increases. This finding is consistent with other studies showing that OOP expenditure on medicines increases with age, with proximity to death resulting in higher medicine expenditure (55, 56). In the context of Saudi Arabia, two plausible reasons explain why increasing age is significantly linked to an increased likelihood of OOP expenditure in medicines. First, aging is often associated with medical complications, including chronic diseases. As a result, in cases where there are no medicines available in public health facilities for such conditions, medicines are acquired from private health facilities through OOP spending (10). Second, polypharmacy constitutes a high proportion of expenditure on medicines, especially among older adults. Aging places individuals at risk of multi-morbidity due to associated physiological and pathological changes and thereby increases the chances of being prescribed multiple medications (57).

In addition, the results showed an increased likelihood of OOP expenditure on medicines at all levels of education compared with that of individuals without primary school education. This finding indicates that individuals at all levels of education may be aware of the importance of health and have acquired more knowledge about healthcare alternatives, including OOP spending on medicines. This conclusion corroborates research performed in other highly developed and literate nations where the majority of the population exhibits a sufficient level of knowledge about their health (58, 59). The expectation was that individuals with higher education levels would spend more OOP on medicines compared to those with lower education due to affordability. Moreover, although healthcare services in the KSA are provided free at the point of use regardless of education level, the difference in OOP expenditure on medicines may reflect the unavailability of some medicines in public facilities (24, 25). The provision of free healthcare services by the KSA to the general public may not be sustainable, especially as the cost of financing healthcare in the Kingdom of Saudi Arabia is exacerbated by the current rapid demographic changes, population aging, epidemiological transition, and increasing prices of medical technology (60).

We also found that suffering from a chronic condition increased the likelihood of OOP expenditure on medicines among the sampled respondents. This finding is in line with earlier published studies in both LMICs and HICs (61–63). For instance, Chen et al. (62) found that the OOP expenditure for patients with chronic diseases was more than three times higher than that of patients without chronic conditions. Excess spending on medicines for chronic illnesses is incessant, and, in the long-term, the compounding effects of OOP costs arising from chronic illnesses—together with lost income—can result in severe financial hardship, leading to catastrophic expenditure on medicines. In the context of the KSA, this finding highlights that public healthcare systems do not necessarily eliminate increased OOP expenditure on medicines.

Average household monthly income was significantly associated with OOP expenditure on medicines, suggesting that individuals earning higher income had a higher probability of OOP spending on medicines. Although this finding corroborates the results of related research in other regions (64, 65), it indicates a low probability of catastrophic expenditure in the KSA. However, it is possible that continued OOP expenditures on medicines can lead to catastrophic health expenditures and impoverishment in the long run, even in households with high monthly incomes.

Marital status and employment status showed negative coefficients in the Tobit model, indicating that married and employed individuals had a lower probability of OOP spending on medicines. These associations can be explained by the existence of medical insurance coverage, which is provided to employed individuals and their families in the country as a benefit package. This is consistent with the findings from other studies showing that medical insurance reduces the probability of OOP expenditure in health (66, 67). Moreover, being married has been significantly associated with medical insurance coverage in Saudi Arabia (22).

Our study has some limitations, which can be explored in future research. First, the measures of health-related variables and estimates of OOP expenditure on medicines are self-reported. As a result, the cost an individual would have spent on medicines is subject to recall bias or incomplete reporting; therefore, a more objective measure of the amount of OOP expenditure needs to be explored. Second, our analyses are cross-sectional and do not consider dynamic changes in OOP expenditure over time. However, this study has several notable strengths. First, it utilizes a large, nationally representative sample, enhancing the generalizability of the findings to the adult population in Saudi Arabia. In addition, by focusing specifically on OOP expenditures on medicines, this study fills a gap in the existing literature, particularly in the context of Saudi Arabia where most of the research in this field to date has focused on general healthcare costs. Furthermore, the use of Tobit regression analysis allows for a nuanced understanding of the factors influencing OOP expenditures, taking into account both zero expenditures and positive spending, which provides a more comprehensive view of the economic burden.

## Conclusion

Age, average household monthly income, education level, and suffering from a chronic condition were significant determinants of OOP expenditure on medicines in the sampled population. The government of the KSA should consider increasing health insurance benefits for groups that are most vulnerable to CHE (i.e., families with low income and high household expenditure, those with a low education level, and those suffering from chronic conditions) due to excessive expenditures on medicines. This study could assist policymakers in providing additional insurance funding to reduce OOP expenditure on medicines.

## Data Availability

The datasets generated and/or analysed during the current study are not publicly available due to privacy, confidentiality, and other restrictions. Access to data can be gained through the General Authority for Statistics in Saudi Arabia via https://www.stats.gov.sa/en.
